# Increased Pan-Type, A1-Type, and A2-Type Astrocyte Activation and Upstream Inflammatory Markers Are Induced by the P2X7 Receptor

**DOI:** 10.3390/ijms25168784

**Published:** 2024-08-13

**Authors:** Keith E. Campagno, Puttipong Sripinun, Lily P. See, Jiaqi Li, Wennan Lu, Assraa Hassan Jassim, Néstor Más Gómez, Claire H. Mitchell

**Affiliations:** 1 Department of Basic and Translational Science, University of Pennsylvania, Philadelphia, PA 19104, USA; 2 Department of Orthodontics, University of Pennsylvania, Philadelphia, PA 19104, USA; 3 Department of Endodontics, University of Pennsylvania, Philadelphia, PA 19104, USA; 4 Department of Chemistry, Department of Basic and Translational Science, University of Pennsylvania, Philadelphia, PA 19104, USA; 5 Department of Basic and Translational Science, Department of Physiology, University of Pennsylvania, Philadelphia, PA 19104, USA

**Keywords:** neuroinflammation, neurotoxic astrocyte polarization, microglia, P2X7, retinal ganglion cells, C3, glaucoma

## Abstract

This study asked whether the P2X7 receptor was necessary and sufficient to trigger astrocyte polarization into neuroinflammatory activation states. Intravitreal injection of agonist BzATP increased gene expression of pan-astrocyte activation markers *Gfap*, *Steap4*, and *Vim* and A1-type astrocyte activation markers *C3*, *Serping1*, and *H2T23*, but also the *Cd14* and *Ptx3* genes usually associated with the A2-type astrocyte activation state and *Tnfa*, *IL1a*, and *C1qa*, assumed to be upstream of astrocyte activation in microglia. Correlation analysis of gene expression suggested the P2X7 receptor induced a mixed A1/A2-astrocyte activation state, although A1-state genes like *C3* increased the most. A similar pattern of mixed glial activation genes occurred one day after intraocular pressure (IOP) was elevated in wild-type mice, but not in P2X7^-/-^ mice, suggesting the P2X7 receptor is necessary for the glial activation that accompanies IOP elevation. In summary, this study suggests stimulation of the P2X7R is necessary and sufficient to trigger the astrocyte activation in the retina following IOP elevation, with a rise in markers for pan-, A1-, and A2-type astrocyte activation. The P2X7 receptor is expressed on microglia, optic nerve head astrocytes, and retinal ganglion cells (RGCs) in the retina, and can be stimulated by the mechanosensitive release of ATP that accompanies IOP elevation. Whether the P2X7 receptor connects this mechanosensitive ATP release to microglial and astrocyte polarization in glaucoma remains to be determined.

## 1. Introduction

Astrocytes can adopt a range of phenotypes and expression profiles in response to neural injury, with classifications distinguished by their distinct patterns of transcriptional alterations [1,2]. The A1- and A2-type forms of astrocyte activation states are associated with neurotoxicity and neuroprotection, respectively, and may contribute to neurodegeneration [3,4]. For example, A1-type astrocytes are implicated in Alzheimer’s disease, Parkinson’s disease, glaucoma, and parasitic infection in the brain [5,6,7]. Astrocyte polarization is also associated with the loss of retinal ganglion cells (RGCs) following the elevation of intraocular pressure (IOP) [3,4,8,9,10]. While distinction between the neurotoxic A1 and neuroprotective A2 astrocytes has received considerable attention, detailed studies suggest astrocyte activation, like microglial activation, is not always binary, and that the particular combination of behaviors and markers is the most accurate way to describe changes in glial inflammation [11,12,13,14]. To limit the pathological consequences for neurons, a better understanding of these inflammatory changes in glial cells and their upstream triggers is required.

The signaling pathways that link astrocyte activation to neuronal damage are emerging. Released lipoparticles containing APOE and APOJ from A1-type-activated astrocytes were found to mediate the downstream effects on neuron health [15]. Signals leading to astrocyte polarization have also been identified, with exposure to cytokines IL-1α, C1q, and TNFα implicated in the activation of neurotoxic astrocytes; neurons were protected and the polarization response induced by IOP elevation was blunted in mice missing *Il1a*, *Tnfa*, and *C1qa* [3,4,8]. However, the upstream mediators initiating the neurotoxic response of increased *Il1a*, *Tnfa*, and *C1qa*, and thus glial activation, following IOP elevation remain largely unknown.

Extracellular ATP is proposed here as a prime candidate responsible for initiating polarized glial activation in many pathological situations involving tissue stretch or swelling, including the elevation of IOP. Mechanical strain releases ATP from many tissues [16], and this released ATP can stimulate the ionotropic P2X7 receptor (P2X7R) to mediate inflammatory responses [17,18,19]. Stimulation of the P2X7 receptor is frequently associated with activation of the NLRP3 inflammasome and the resulting release of cytokines like IL-1β [19,20,21,22], although receptor stimulation also induces release of other inflammatory signals [23]. The P2X7 receptor is expressed on RGCs, optic nerve head astrocytes, and retinal microglia, and elevation of IOP can trigger a mechanosensitive ATP release and stimulation of the receptor [24]. Whether the P2X7 receptor can lead to astrocyte polarization is unknown, however.

This study tests whether the P2X7 receptor can increase the expression of genes associated with the pan-, A1-, or A2-astrocyte activation state in the retina. Intravitreal administration of P2X7 receptor agonist BzATP upregulated expression of *Tnfa*, *C1qa* and *Il1a*, as well as genes associated with A1- and A2-specific astrocyte polarization. A parallel response was induced by IOP elevation in wild-type, but not P2X7^-/-^, mice, suggesting the receptor was also necessary for early transcriptional changes. Overall, this identifies a key role for the P2X7 receptor in the astrocyte activation patterns linked to neurotoxicity.

## 2. Results

### 2.1. Injection of P2X7 Receptor Agonist BzATP Increases Expression of Genes Associated with A1-Type Astrocyte Inflammation

To test the hypothesis that P2X7 receptor stimulation was upstream from astrocyte activation, initial experiments examined whether direct stimulation of the P2X7 receptor with agonist BzATP was sufficient to increase the expression of genes associated with astrocyte activation states in the retina. BzATP (250 μM) was injected intravitreally with saline injected into the contralateral eye, and retinas were removed 24 h later [25]. As genes associated with the differential astrocyte activation states have already been identified [8], gene expression was compared with qPCR.

**Figure 1:**
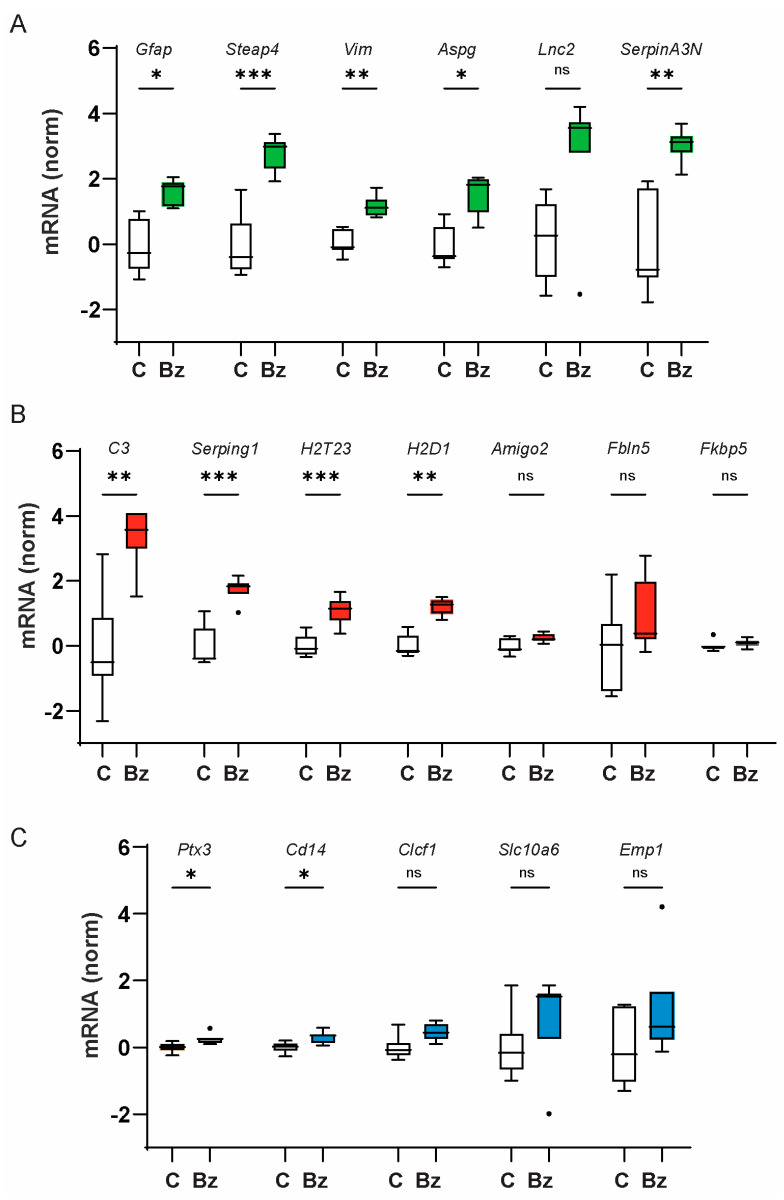
P2X7 agonist BzATP upregulates mixed astrocyte activation markers. Expression of mRNA associated with astrocyte activation states in C57Bl/6J mouse retinas extracted 24 h after intravitreal injection of BzATP (Bz; 250 µM) or saline controls (C). (**A**) Expression of mRNA for pan-astrocyte activation markers *Gfap*, *Steap4*, *Vim*, *Aspg*, and *SerpinA3N* was increased. Throughout the Figure, data were analyzed with a one-way ANOVA with the Šidák multiple comparisons test; * = *p* < 0.05, ** = *p* < 0.01, *** = *p* < 0.001, and ns = not significant; *n* = 7 pairs. Throughout the manuscript, mRNA expression is expressed as ΔΔCT and normalized to the mean value for control eyes. Box and whisker plots were generated based on Tukey analysis, where the box indicates median ± 25th and 75th percentile, and dots show outliers. (**B**) BzATP injection also increased the expression of genes associated with the A1 activation state *C3*, *Serping1*, *H2T23*, and *H2D1*. (**C**) The rise in genes associated with the A2 activation state *Ptx3* and *Cd14* was significant but small.

BzATP injection significantly upregulated expression of genes associated with pan-reactive astrocytes (*Gfap*, *Steap4*, *Vim*, *SerpinA3N*, and *Aspg*) as compared to saline-injected eyes (Figure 1A). BzATP injection also elevated expression of the A1-activation-state genes *C3*, *Serping1*, *H2T23*, and *H2D1* (Figure 1B). Levels of *C3* increased the most, corresponding to a 20-fold rise following drug injection when converted to relative expression. The rise in *CD14* and *Ptx3*, genes associated with the A2 activation state, was small but significant (Figure 1C). BzATP did not significantly change the expression of other genes associated with the A1 (*Amigo2*, *Fbln5*, *Fkbp5*) or A2 activation state (*Clcf1*, *Emp1*, *Slc10a6*). Overall, the pattern of gene expression found after intravitreal injection of BzATP was consistent with astrocyte activation, with genes associated with pan-, A1-, and, to a smaller extent, A2-astrocyte activation states.

### 2.2. P2X7 Receptor Agonist BzATP Increases the Expression of Genes Upstream of Astrocyte Inflammation

**Figure 2:**
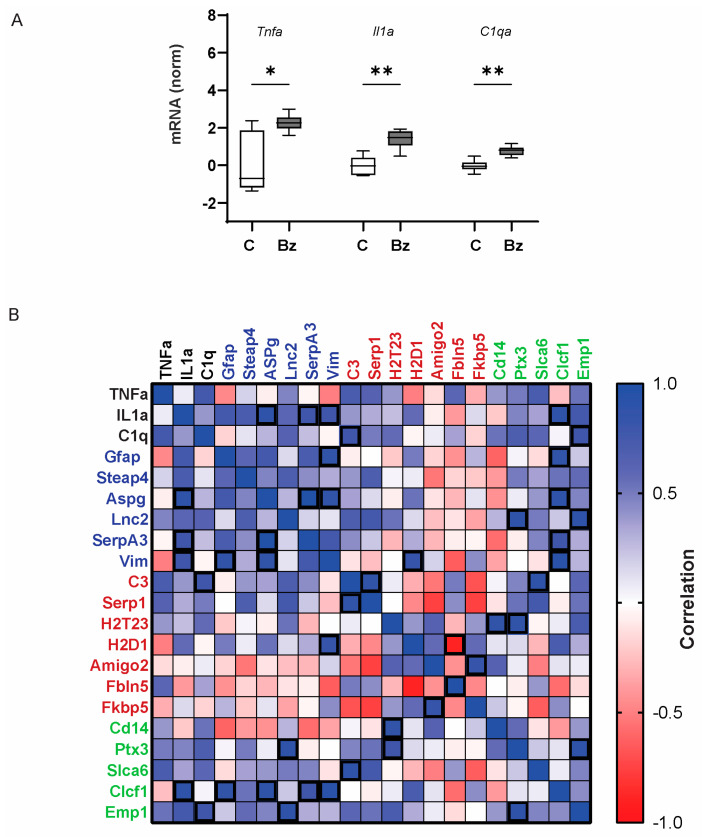
Correlation of gene changes induced by BzATP. Expression of mRNA message in C57Bl/6J mouse retinas extracted 24 h after intravitreal injection of BzATP (Bz, 250 µM) or saline controls (C). (**A**) Expression of mRNA for *Tnfa*, *Il1a*, and *C1qa* was increased in eyes injected with BzATP as compared to the contralateral eyes injected with equal volumes of saline. One-way ANOVA with Šidák multiple comparisons test, *n* = 7 pairs; * = *p* < 0.05, ** = *p* < 0.01. (**B**) Spearman’s correlation between changes in all genes induced by injection of BzATP as compared to levels in the contralateral control retinas. Blue squares indicate a positive correlation and red a negative correlation, with the intensity of color an index of the correlation magnitude. Genes named in black are associated with microglia, green with pan-, red with A1-, and blue with A2-associated activation markers. Correlations with *p* < 0.05 are marked with a thick cube.

As cytokines TNF-α, IL-1α, and C1qa are implicated in promoting A1-type neurotoxic astrocyte inflammation [8,26], the effect of BzATP injection on the expression of *Tnfa*, *Il1a*, and *C1qa* was also examined (Figure 2A). Intravitreal injections of BzATP increased the expression of *Tnfa*, *Il1a* and *C1qa* as compared to saline-injected eyes. When combined with the results from Figure 1, these results suggest stimulation of the P2X7 receptor with BzATP may be sufficient to increase the expression of multiple glial activation markers.

The overall pattern of changes in all 21 genes induced by BzATP injection was examined by analyzing the relationships between them with a Spearman’s correlation (Figure 2B). The analysis showed that BzATP led to substantial changes in the expression of many genes associated with glial activation that were significantly correlated with one another. Some of the correlations were expected, such as the significant correlation between the expression of *Vim* and *Il1a, Gfap*, and *Aspg*. However, the A1-associated marker *H2T23* was significantly correlated with the expression of *Ptx3* and *Cd14* genes linked to A2-type astrocyte polarization. Changes in *Il1a* expression correlated well with changes in the pan-astrocyte activation genes and several of the A1-associated and A2-associated genes, but not other A1- and A2-associated genes. A similar pattern was found for the correlation with multiple genes including *Aspg, Steap*, and *Lnc2*. In total, the correlation analysis strengthens the link between the P2X7 receptor and upregulation of multiple genes associated with glial activation, while suggesting either the response or the markers are not as polarized as often assumed.

### 2.3. P2X7 Receptor Contributes to Neurotoxic Astrocyte Activation after IOP Elevation

**Figure 3:**
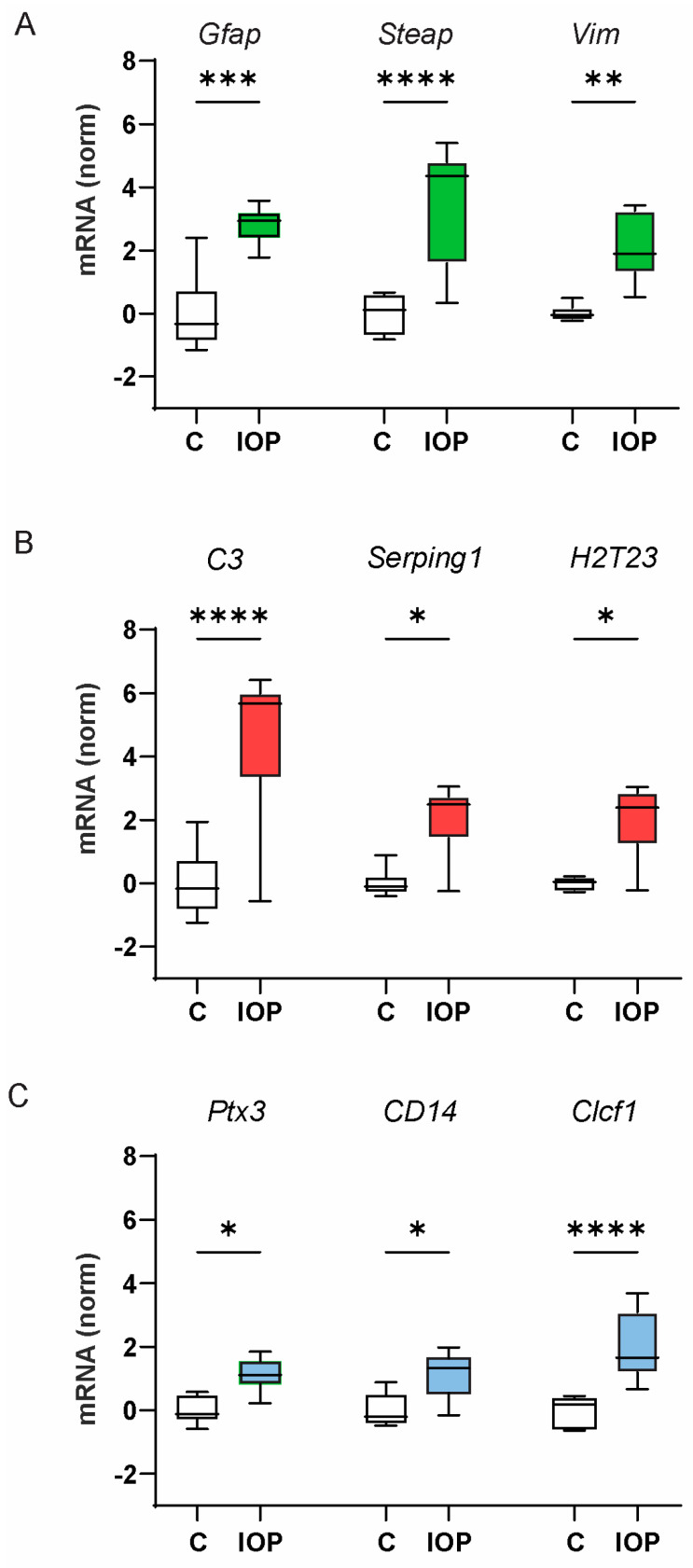
A rise in mixed-astrocyte activation markers accompanied IOP elevation in C57Bl/6J mice. (**A**) Transient elevation of IOP in C57Bl6/J mice (IOP) increased expression of mRNA for genes associated with pan-astrocyte activation, *Gfap, Steap4*, and *Vim*, as compared to the unpressurized contralateral control eye (C). mRNA for genes associated with A1-astrocyte activation, *C3^+^, Serping1^+^*, and *H2T23*, (**B**) and with the A2-astrocyte activation state, *Ptx3, Cd14*, and *Clcf1*, (**C**) were also increased by IOP elevation. One-way ANOVA with Šidák multiple comparisons test, *n* = 7 pairs; * = *p* < 0.05, ** = *p* < 0.01, *** = *p* < 0.001, **** = *p* < 0.0001.

Elevation of IOP is a major risk factor for vision loss and retinal ganglion cell injury in glaucoma, and a rise in IOP has been associated with an increase in the expression of neurotoxic astrocyte markers and RGC loss in models of glaucoma [3,4]. To separate the initial effects of tissue stretch from the downstream cascades associated with cell death, a non-ischemic elevation of IOP was induced for 4 h, and gene expression analyzed from retinal tissue was obtained 22–24 h later. Sham injections did not change gene expression (Figure S1). The three genes associated with each state showing the largest response above were selected for analysis. A significant rise in the expression of pan-reactive astrocyte transcripts *Gfap*, *Steap4*, and *Vim* was detected in the retinas from C57Bl/6J mice exposed to the transient IOP elevation (Figure 3A). The expression of *C3*, *Serping1*, and *H2T23*, transcripts associated with the A1-astrocyte activation state, were also elevated (Figure 3B). An increase in the expression of *Cd14*, *Ptx3*, and *Clcf1* genes usually associated with the A2 state was also detected (Figure 3C). Spearman’s correlation analysis of the changes in the glial activation genes induced following IOP elevation suggests groupings similar to those found after BzATP injection (Figure S2).

### 2.4. P2X7 Receptor Is Necessary for the Increased Expression of Tnfa and Il1a Following IOP Elevation

**Figure 4:**
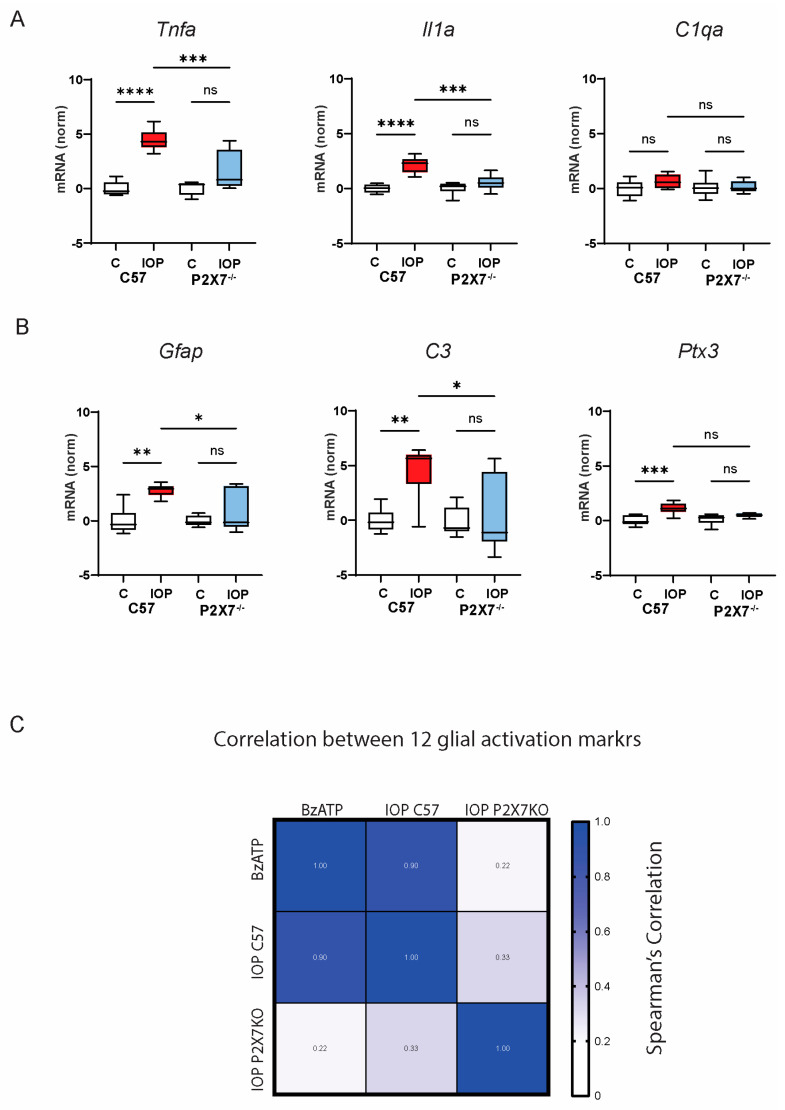
P2X7R is required for the rise in many glial activation markers after IOP elevation. (**A**) Expression of *Tnfa* and *Il1a* increased in retinas from C57Bl/6J mice (C57) but not in P2X7^-/-^ mice exposed to a transient IOP elevation (IOP; red for C57, blue for P2X7^-/-^ mice). Expression normalized to normotensive control eye. (**B**) IOP elevation increased *Gfap, C3*, and *Ptx3* expression in retinas from C57Bl/6J mice, but not in P2X7^-/-^ mice. One-way ANOVA with Šidák multiple comparisons test, *n* = 6 pairs. * = *p* < 0.05, ** = *p* < 0.01, *** = *p* < 0.001, **** = *p* < 0.0001, ns = not significant. (**C**) Spearman’s correlation analysis calculated for changes in mean expression of *Tnfa, Il1a, C1qa, Gfap, Steap, Vim, C3, Serping1 H2T23, Ptx3, Cd14,* and *Clcf1* following BzATP injection; transient IOP elevation in C57Bl/6J mice (IOP C57) or P2X7^-/-^ mice (IOP P2X7KO). The Spearman’s coefficient is given in boxes; darker blue indicates a closer correlation. Data indicate that the patterns of gene expression changes with BzATP and IOP elevation in C57 mice were closely correlated with each other (*p* = 0.00015), but not with changes in P2X7^-/-^ mice following IOP elevation. The correlation was based on the mean values for each gene from 7, 6, and 6 mice for BzATP, IOP C57, and IOP P2X7KO conditions, respectively, as compared to the contralateral control retinas.

To determine whether the P2X7 receptor contributed to the IOP response, transient IOP was performed in P2X7^-/-^ mice. IOP elevation in P2X7^-/-^ mice did not lead to a significant rise in any of the astrocyte activation markers that were affected in the retina of wild-type mice, whether associated with pan-astrocyte activation, A1 activation, or the A2 activation state, with the exception of Clcf1 (Figure S3). The ability of IOP elevation to increase retinal expression of *Tnfa*, *Il1a*, and *C1qa* was compared directly in C57Bl/6J and P2X7^-/-^ mice to examine whether the receptor was required for these genes thought to be upstream of astrocyte activation. Comparative analysis suggested transient IOP elevation increased the expression of *Tnfa* and *Il1a* in the retinas from C57Bl/6J mice, but not in P2X7^-/-^ mice (Figure 4A). Direct comparison of the change in astrocyte gene expression between C57Bl/6J and P2X7^-/-^ mice provided similar results; the expression of *Gfap*, *C3*, and *Ptx3* was significantly increased following IOP elevation in retinas from C57Bl/6J mice but not P2X7^-/-^ mice (Figure 4B).

While the elevation of IOP did raise markers for pan-, A1, A2, and microglial activation, the reduced expression in mice missing the P2X7 receptor strongly suggested the P2X7 receptor was required for the response. This was similar to the response induced by the P2X7 receptor agonist BzATP. To determine whether the expression changes found following BzATP injection and IOP elevation were similar, a Spearman’s correlation analysis was performed, using the mean expression changes for 12 glial markers: *Tnfa*, *Il1a* and *C1qa*; *Gfap*, *Steap4*, and *Vim*; *C3*, *Serping1*, and *H2T23*; and *Cd14*, *Ptx3*, and *Clcf1.* There was a close correlation between the pattern of gene changes induced by BzATP injection and IOP elevation (*p* = 0.00015, correlation coefficient = 0.902). However, the response in P2X7^-/-^ mice exposed to IOP elevation was not correlated with the expression changes found in either BzATP-treated eyes (*p* = 0.480, correlation coefficient = 0.224) or wild-type mice with IOP elevation (*p* = 0.299, correlation coefficient = 0.326). Together the correlation analysis, provides powerful statistical backing for the hypothesis that the rise in glial activation genes in response to IOP elevation is largely dependent upon the P2X7 receptor.

## 3. Discussion

Glial cells are increasingly recognized for their roles in neurodegeneration, with inflammatory neurotoxic astrocytes implicated in the response to multiple stresses [8,26,27]. The signaling pathways that initiate these events and coordinate the pathological responses are less well understood, however. The data above implicate the P2X7 receptor in the upregulation of the genes associated with astrocyte activation, and of the genes *Tnfa* and *Il1a*, presumed to be upstream of this astrocyte activation [8]. The ability of P2X7 receptor agonist BzATP to increase gene expression suggests receptor stimulation is itself sufficient to activate transcription, while the reduced response in P2X7^-/-^ mice exposed to IOP elevation as compared to wild-type mice suggests the receptor is also necessary for the increased glial activation that accompanies elevated IOP. Given the documented links between IOP elevation and the release of the P2X7 receptor agonist ATP [28,29], these results may provide a broader context to explain these pathological neural/glial interactions.

### 3.1. The P2X7 Receptor Is Sufficient and Necessary for Glial Activation

The response to intravitreal injection of BzATP supports the role of the P2X7 receptor in astrocyte activation. The P2X7 receptor agonist upregulated the expression of complement component genes *C3* and *Serping1*, as well as Major Histocompatibility Complex 1 (MHC I) genes *H2T23* and *H2D1*. However, genes associated with the A2-astrocyte state were also elevated. Transcription of glial activation genes was increased in a similar pattern after IOP elevation in wild-type, but not P2X7^-/-^, mice. The correlation in the degree of change in the 12 genes in response to both BzATP injection and IOP elevation supports this, particularly as the pattern of gene change in P2X7^-/-^ mice exposed to IOP elevation was not significantly correlated with either group (Figure 4C). Together, this strongly suggests that the P2X7 receptor is both sufficient (with BzATP) and necessary (in contrast with the knockout) for the rise in these glial activation markers.

It should be noted that although BzATP is frequently used as a P2X7 receptor agonist, it can also act at P2X1, P2X2, and P2X3 receptors and the heteromeric P2X2/P2X3 receptors [30]. Involvement of these receptors in the response to BzATP cannot be ruled out, and their increased sensitivity to lower ATP levels may lead to activation at more moderate concentrations. However, the reduced elevation of *Gfap*, *C3*, *Tnfa*, and *Il1a* following IOP elevation in P2X7^-/-^ mice in Figure 4 (and Figure S3) supports a specific contribution of the P2X7 receptor. The effects of P2X7 receptor stimulation following IOP elevation have been confirmed with specific antagonists by multiple investigators [31,32,33,34,35], further supporting a role for the receptor.

### 3.2. Astrocyte Activation States: Mixed States or Mixed-Up Markers?

Direct stimulation of the P2X7 receptor with BzATP significantly increased expression of five markers associated with the pan-astrocyte activation state (*Gfap*, *Steap4*, *Vim*, *Aspg*, *SerpinA3N*), four out of seven of the genes associated with the A1-astrocyte activation state (*C3*, *Serping1*, *H2T32*, *H2D1*), and two of five genes associated with the A2 activation state (*Ptx3* and *Cd14*). The examination of the responses using a Spearman’s correlation analysis enabled the pattern of linked changes to be evaluated; this pattern suggests that either the P2X7 receptor initiates both A1- and A2-type activation states in retinal astrocytes, or that the assignment of “marker” genes is not as predictive as often assumed. The strict classification of A1- and A2-astrocyte activation states is unlikely to apply in most neural systems, with the pattern of astrocyte polarization being contextual and no longer considered binary [12,36]. Whether the data reflect activation of a mixed activation state, or of markers that are not completely binary, is hard to predict with certainty, however. For example, the rise in the *Cd14*, *Ptx3*, and *Clcf1* genes associated with the A2 activation state could reflect an involvement of the A2 state in the early response to IOP elevation, or just a misclassification of the markers. Regardless of the chosen definition, the pattern of gene upregulation is most consistent with the P2X7 receptor leading to a clear rise in the astrocyte activation state. It should be noted that the comparison of data for 21 genes from all seven sets of saline versus BzATP-injected retinas adds rigor to the correlation; unlike RNAseq data, the results have already been confirmed with PCR.

### 3.3. The P2X7 Receptor Is Sufficient and Necessary for Activation of Tnfa and Il1a

The analysis clearly supports a role for the P2X7 receptor in the elevation of *Tnfa* and *Il1a*, with an increase following both BzATP injection and IOP elevation. Levels of *C1qa* were significantly increased following BzATP injection, although the smaller rise following transient IOP elevation was not significant.. As these genes are considered to be upstream from the A1-astrocyte activation [8], this supports a model where stimulation of the P2X7 receptor could initiate the overall glial activation response (see Graphical Abstract).

While the role of the P2X7 receptor in the upregulation of *Tnfa* and *Il1a* is clear, the cellular location of this response is uncertain. Microglial cells were thought to be the primary source of these released inflammatory signals; in vitro polarization of microglia with lipopolysaccharide led to the release of TNF-α, IL-1α, and C1q, and this secretome in turn polarized cultured astrocytes into an A1 neurotoxic phenotype [8]. However, ablation of microglia with PLX5622 and subsequent application of the optic nerve crush model did not rescue the neuronal loss, suggesting a role for cells in addition to microglia [8]. This agrees with preliminary data suggesting that only some of the IL-1α and TNFα expression is associated with microglia. Previous reports suggest the rise in TNFα protein expression accompanying IOP elevation is detected in RGCs [37].The rise in neural TNFα protein expression following BzATP injection is currently being investigated, but it does engender caution about attributing the TNFα source.

### 3.4. Physiological Implications

The observations above implicate the P2X7 receptor in the activation of *Tnfa*, *Il1a*, and multiple astrocyte activation markers. The P2X7 receptor may have particular importance in conditions associated with tissue stretch such as IOP elevation, and increased ATP levels are associated with chronic ocular hypertension models in rats, mice, and non-human primates, and in humans with chronic glaucoma [24,28]. While the transient model of IOP elevation used in this study does not emulate the chronic condition, it does enable specific examination of an early response to IOP elevation, before the inflammation and cell death produce secondary responses. The analysis of transcriptional changes provides a snapshot of the initial responses to IOP elevation in vivo. The early timepoint also minimizes the likelihood of infiltration of peripheral monocytes into the retina. The findings are supported by reports using more chronic models of ocular hypertension that identify a contribution of inflammatory genes [38,39].

The development of neurotoxic astrocytes is increasingly recognized in neuroinflammatory events; astrocyte reactivity is implicated in glaucoma, while astrocyte polarization following microglial activation is emerging as a pivotal component of this reactivity [3,4,8,10,40,41]. The development of astrocyte activation in the contralateral retina was recently shown to require pannexin 1 [42]; as pannexin 1 is a conduit for mechanosensitive ATP release, this supports a role for extracellular ATP in the pathological developments.

Complement factor 3 (C3) is recognized as a key marker for neuroinflammation in many neurodegenerative diseases [43,44,45], and has recently been implicated in glaucoma [46,47,48,49]. In the present study, *C3* expression rose more than any other astrocyte activation marker in response to either BzATP injection or IOP elevation. While IOP elevation increased C3 levels in wild-type mice, there was no response in P2X7^-/-^ mice, implicating the receptor in rising C3 levels. The rise in *C3* expression following BzATP injections was significantly correlated with *C1qa*, *Serp1*, and *Clcf1*, while preliminary immunohistochemistry indicated that the retinal localization was clearly astrocytic. The development of neurotoxic astrocytes and the expression of C3 in the injured optic nerve was recently linked to soluble adenylate cyclase [49]. Future studies will determine whether stimulation of the P2X7 receptor activates soluble adenylate cyclase in the retina and optic nerve, but a connection is feasible given that the opening of the pore in the ionotropic P2X7 receptor channel leads to a rapid influx of Ca^2+^ [50], and Ca^2+^ increases activity of soluble adenylate cyclase [51,52].

While the ability of polarized neurotoxic astrocytes to kill retinal ganglion cells in experimental models has been established [4,8,15], the overall effect of P2X7 receptor stimulation on retinal health may well be context-dependent. The receptor was originally referred to as the “death receptor” [53], but its expression on healthy, long-lived cells such as retinal neurons suggests it does far more than just kill cells [54]. The P2X7 receptor can act as a protective scavenger receptor in the absence of agonists [55], while stimulation of isolated RGCs triggers the release of many cytokines including IL-3, with IL-3 itself being protective [56]. Whether glial activation inducted by the P2X7 receptor leads directly to neuronal death, or whether the consequences are more nuanced, remains to be determined.

### 3.5. Summary

In summary, this study suggests the retinal P2X7 receptor leads to activation of microglia and astrocytes. Receptor stimulation increased the expression of genes associated with pan-, A1-, and A2-type astrocyte activation. Correlation analysis has identified a parallel rise in some, but not all, genes associated with the astrocyte activation states. Elevation of IOP in wild-type but not P2X7^-/-^ mice induced a similar change in retinal gene expression, indicating a role for the receptor in the neuroinflammatory response. As excessive ATP is released with IOP elevation, the P2X7 receptor may provide an upstream trigger, linking increased pressure with neurotoxic astrocyte activation.

## 4. Materials and Methods

### 4.1. Animal Care and Use

All procedures were performed in strict accordance with the National Research Council’s “Guide for the Care and Use of Laboratory Animals” and were approved by the University of Pennsylvania Institutional Animal Care and Use Committee (IACUC, protocol #803584). All animals were housed on a 12:12 light:dark cycle in temperature-controlled rooms with food and water ad libitum. Mice (C57Bl/6J wild-type and P2XR7^−/−^) were obtained from Jackson Laboratories, Bar Harbor, ME, using strains #000664 and #005576, respectively. Mice were genotyped regularly to confirm the knockout (Figure S3D).

### 4.2. Intravitreal Injections

Intravitreal injections were performed as previously described [57]. In brief, C57Bl/6J mice were anesthetized with 1.5% isofluorane and subsequently injected under a dissecting microscope using a micropipette attached to a microsyringe (Drummond Scientific Co., Broomall, PA, USA). Injections consisted of sterile Balanced Saline Solution (Teknova, Hollister, CA, USA) as a control or with Benzoylbenzoyl-ATP (BzATP, #B6396, Sigma Aldrich, St. Louis, MO, USA, 200–250 μM). A total of 1.5 μL was injected approximately 0.5 mm posterior to the limbus into the superior nasal region of the vitreous cavity. Retinas were isolated 24 h later for RNAextraction. The concentration of BzATP was chosen based upon previous trials [25,33]. Eyes were carefully inspected and any mouse showing evidence of lens damage was removed from the study.

### 4.3. Transient Elevation of IOP

Transient elevation of IOP was performed in adult mice of both sexes using modifications of the controlled elevation of IOP procedure developed by the Morrison group [58], as previously described [25]. Briefly, mice were anesthetized with 1.5% isoflurane after receiving 2 mg/kg meloxicam. Proparacaine (0.5%) and tropicamide (0.5–1%) were administered, then the anterior chamber of one eye was cannulated with a 33-gauge needle attached to polyethylene tubing (PE 50; Becton Dickinson, Franklin Lakes, NJ, USA) connected to a 20 mL syringe filled with sterile PBS. The reservoir was elevated to the appropriate height to increase IOP to 57.0 ± 3 mmHg. Inspection indicated that retinal blood flow was maintained throughout, avoiding acute ischemia, although some reduction in blood flow cannot be ruled out. IOP was returned to baseline after 4 h and 0.5% erythromycin was applied to the cornea. The contralateral eye without cannulation served as a normotensive control. Sham injection did not induce an inflammatory response. Retinal tissues were isolated 22–24 h after elevation of IOP, as previous studies have pointed to robust gene expression at this time point [25].

### 4.4. Quantitative PCR

Evaluation of RNA expression utilizing qPCR was based on published methods [33]. In brief, the retina was homogenized in TRIzol reagent (#15596018, Invitrogen, Waltham, MA, USA); RNA was purified with an RNeasy mini kit (#79254, Qiagen, Inc., Germantown, MD, USA) and converted to cDNA using the High Capacity cDNA Reverse Transcription Kit (#4368814, Applied Biosystems Corp., Foster City, CA, USA). Gene expression was assayed using PowerUp Sybr Green (#A25742, Applied Biosystems Corp.) on the Quant Studio 3 Real-Time PCR system (Applied Biosystems Corp.). Primers are listed in Table 1.

### 4.5. Data Analysis and Materials

All materials were obtained from Sigma Aldrich Corp. (St. Louis, MO, USA) unless otherwise noted. Data are displayed as mean ± standard deviation. Statistical analysis was performed using GraphPad Prism software version 9.0.0 (GraphPad Software, Inc. San Diego, CA, USA). Significant differences were assessed with a one-way ANOVA followed by a Šidák multiple comparisons test. Data are expressed with box and whisker plots based upon the Tukey analysis approach. Results returning *p* < 0.05 were considered significant. All 88 data sets were tested for normality using both the Shapiro–Wilk and Kolmogorov–Smirnov tests, and all but 6 passed. While all ANOVA tests were significant, Gaussian distribution was not assumed; the nonparametric Spearman’s analysis was thus chosen to evaluate correlation between gene changes across samples, or to compare patterns of expression change induced by BzATP and IOP elevation.

## Figures and Tables

**Table 1:** Primers used for qPCR.

Gene Name	Genbank Accession	Primer (F: 5′–3′; R: 3′–5′)	Size (bp)
*Tnfa*	NM_013693.3	F: AAATGGCCTCCCTCTCATCAG R: GTCACTCGAATTTTGAGAAGATGATC	73
*Il1a*	NM_010554.4	F: CAACGTCAAGCAACGGGAAG R: AAGGTGCTGATCTGGGTTGG	126
*C1qa*	NM_007572.2	F: GAAGGGCGTGAAAGGCAATC R: CAAGCGTCATTGGGTTCTGC	86
*Gfap*	NM_001131020.1	F: CCTGCCAGCTCTCCCT R: AAAGGTGTGGCTGAAATGCG	216
*Steap4*	NM_054098.3	F: CCCGAATCGTGTCTTTCCTA R: GGCCTGAGTAATGGTTGCAT	262
*Vim*	NM_011701.4	F: GATGGCCCTGGACATTGAGA R: TTGAGTGGGTGTCAACCAGAG	146
*Lcn2*	NM_008491.1	F: CCAGTTCGCCATGGTATTTT R: CACACTCACCACCCATTCAG	206
*SerpinA3N*	NM_009252.2	F: GCTGGCTGGTTTCAGCTCT R: ATCCATTCCCAACGTGCCAT	127
*Aspg*	NM_001081169.1	F: GCTGCTGGCCATTTACACTG R: GTGGGCCTGTGCATACTCTT	133
*C3*	NM_009778.3	F: TTCCTTCACTATGGGACCAGC R: CTCCAGCCGTAGGACATTGG	127
*Serping1*	NM_009776.3	F: ACAGCCCCCTCTGAATTCTT R: GGATGCTCTCCAAGTTGCTC	299
*H2D1*	NM_010380.3	F: TCCGAGATTGTAAAGCGTGAAGA R: ACAGGGCAGTGCAGGGATAG	204
*H2T23*	NM_010398.3	F: GGACCGCGAATGACATAGC R: GCACCTCAGGGTGACTTCAT	212
*Amigo2*	NM_178114.4	F: GAGGCGACCATAATGTCGTT R: GCATCCAACAGTCCGATTCT	263
*Fkbp5*	NM_010220.4	F: TATGCTTATGGCTCGGCTGG R: CAGCCTTCCAGGTGGACTTT	194
*Fbln5*	NM_001361987.1	F: CTTCAGATGCAAGCAACAA R: AGGCAGTGTCAGAGGCCTTA	281
*CD14*	NM_009841.4	F: GGACTGATCTCAGCCCTCTG R: GCTTCAGCCCAGTGAAAGAC	232
*Ptx3*	NM_008987.3	F: AACAAGCTCTGTTGCCCATT R: TCCCAAATGGAACATTGGAT	147
*Clcf1*	NM_019952.5	F: CTTCAATCCTCCTCGACTGG R: TACGTCGGAGTTCAGCTGTG	176
*Emp1*	NM_010128.4	F: GAGACACTGGCCAGAAAAGC R: TAAAAGGCAAGGGAATGCAC	183
*Slc10a6*	NM_029415.2	F: GCTTCGGTGGTATGATGCTT R: CCACAGGCTTTTCTGGTGAT	217
*GAPDH*	NM_017008	F: TCACCACCATGGAGAAGGC R: GCTAAGCAGTTGGTGGTGCA	169

## Data Availability

Data is contained within the article and Supplementary Materials.

## Supplementary Materials

The following supporting information can be downloaded at: https://www.mdpi.com/article/10.3390/ijms25168784/s1.
