# Supplementary material for: Increased Pan-Type, A1-Type, and A2-Type Astrocyte Activation and Upstream Inflammatory Markers Are Induced by the P2X7 Receptor
Source: Int J Mol Sci. 2024 Aug 13;25(16):8784. doi: 10.3390/ijms25168784 (PMC11354399; doi:10.3390/ijms25168784)
Supplement: Supplementary file 1 [file ijms-25-08784-s001.zip › ijms-2997563-supplementary.pdf]

## Supplemental Figures

Figure S1. Negative control for needle insertion in transient IOP elevation model.

Figure S2. Spearman's correlation for gene changes with IOP elevation in wild-type mice.

Figure S3. IOP elevation and genes in $P2X7^{-/-}$ mice.

**Figure S1**

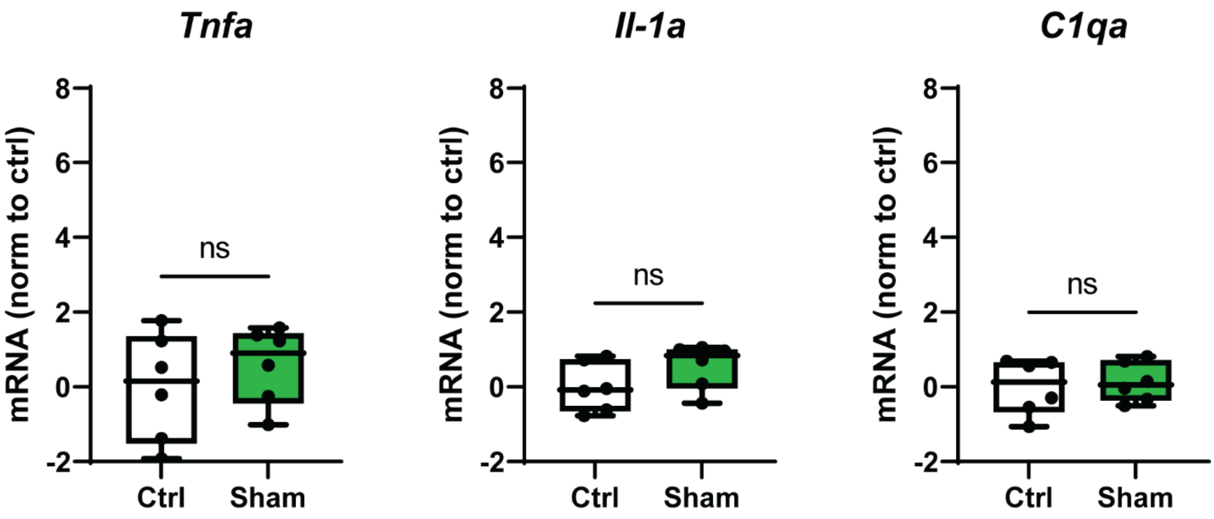


**Figure S1.** Negative control showing the effect of sham needle insertion on expression of *Tnfa*, *Il1a,* and *C1qa*. There was no significant difference in expression. Paired t-test, n=6.

## Figure S2

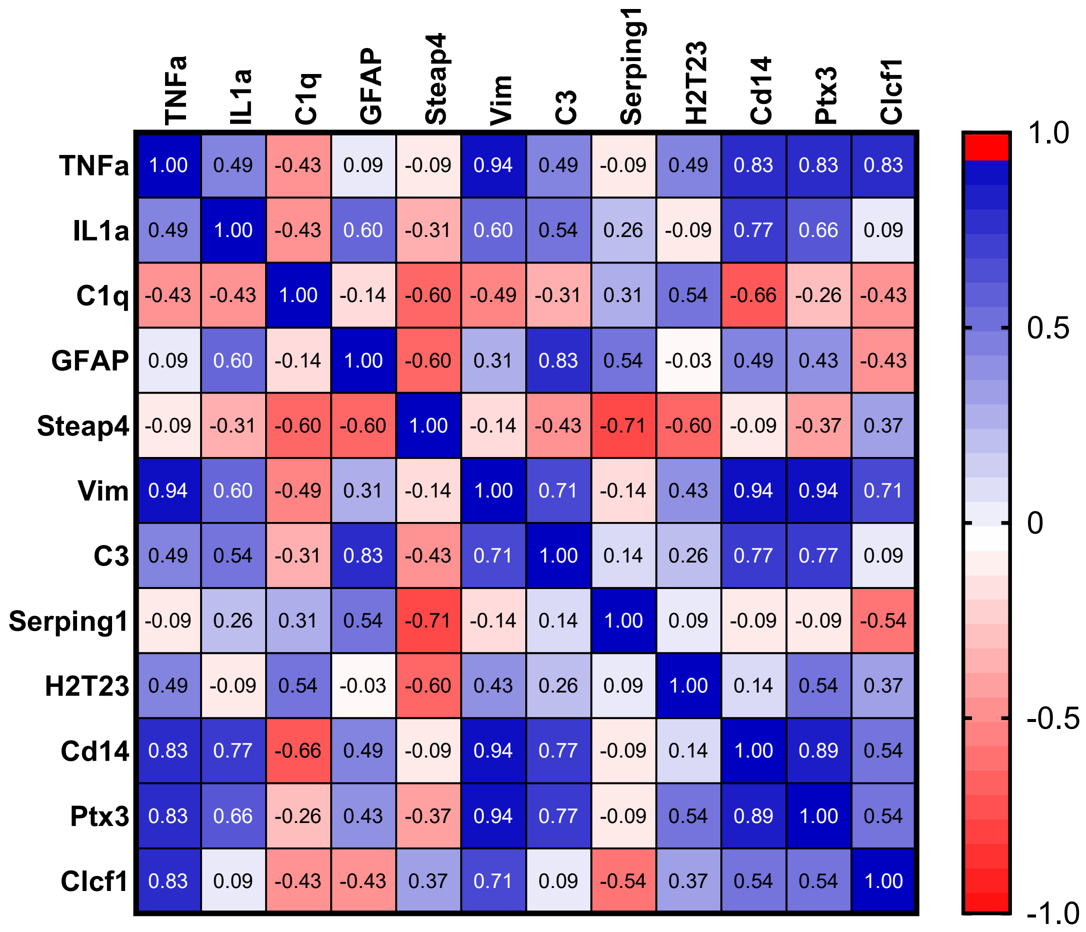


**Figure S2.** Spearman's correlation analysis of the changes between glial activation genes in response to IOP elevation compared to contralateral normotensive controls. Blue squares indicate a positive correlation, and red represents a negative correlation, with the intensity of color indicating an index of the correlation magnitude. Numbers represent the correlation coefficient.

# Figure S3

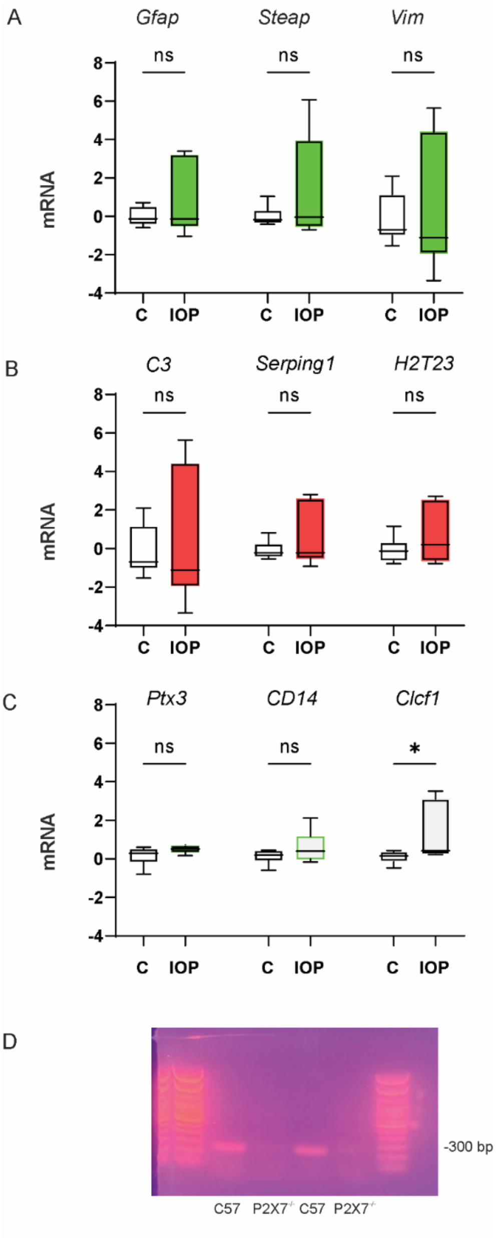


**Figure S3.** Transient elevation of IOP in P2X7-/- mice (IOP) did not significantly change expression of mRNA for genes associated with the following astrocyte activation states as compared to the unpressurized contralateral control eye (C). A. Pan-astrocyte activation *Gfap*, *Steap4*, and *Vim*. B. A1-astrocyte activation *C3, Serping1*, and *H2T23;* C. *Ptx3 and Cd14* genes are associated with the A2-astrocyte activation state. However, only *Clcf1* expression was increased by IOP elevation. One-way ANOVA with Šidák multiple comparisons test, n=6 pairs. * = p<0.05, ns = not significant. D. Genotyping PCR gel for two wildtype (C57) and two P2X7 knockout mice (P2X7-/-) confirming the absence of the PCR product for the P2X7 receptor in knockout mice.
